# Somatic yoga therapy for functional neurological disorder: feasibility randomised controlled trial

**DOI:** 10.1192/bjo.2026.12003

**Published:** 2026-06-02

**Authors:** Emily Kennedy-Barnes, L. S. Merritt Millman, Yasmine Basamh, Anel Duarte, Jenna Pacelli, John Hodsoll, Susannah Pick

**Affiliations:** Department of Psychological Medicine, Institute of Psychiatry, Psychology and Neuroscience, King’s College London, London, UK; Biostatistics and Health Informatics Department, Institute of Psychiatry, Psychology and Neuroscience, King’s College London, London, UK

**Keywords:** Yoga, functional neurological disorder, intervention, feasibility, randomised controlled trial

## Abstract

**Background:**

Functional neurological disorder (FND) is characterised by disabling motor, sensory and/or seizure symptoms. Contemporary models highlight the possible involvement of altered affective and autonomic regulation and interoceptive processing in FND. However, accessible, mechanism-informed interventions remain limited.

**Aims:**

This study sought to examine the feasibility, acceptability and possible benefits of tailored somatic yoga for people with FND, aiming to directly target altered emotional and bodily regulation in this group.

**Method:**

This single-site, two-arm randomised feasibility trial allocated adults with FND to 6 weeks of either somatic yoga (weekly remote sessions plus home practice manual) or a music-based relaxation control. Feasibility outcomes included recruitment, retention, adherence and acceptability. Secondary outcomes, assessed at baseline, week 3, week 6 and 3-month follow-up, included FND symptom burden, psychological symptoms (anxiety, depression, dissociation), general functioning, health-related quality-of-life, interoceptive awareness and autonomic symptoms.

**Results:**

Seventy-six enquiries were received. Thirty participants consented (100%), 27 were randomised (90%), 23 commenced the trial (77%) and 21 completed it (70%). Recruitment and retention targets were met, with 100% retention in the yoga arm. Adherence was high across both groups, although digital logging of home practice posed usability challenges. Exploratory analyses indicated large effect sizes for self-reported FND symptom improvement and interoceptive awareness in the yoga group, with effects sustained at follow-up.

**Conclusions:**

Individually delivered somatic yoga was found to be feasible, acceptable and safe for people with FND. Our results also suggest potential benefits of somatic yoga for FND symptom improvement and interoceptive awareness, supporting progression to a fully powered trial.

Functional neurological disorder (FND) is a prevalent and disabling neuropsychiatric condition characterised by motor symptoms, sensory alterations, seizures or cognitive symptoms that are inconsistent with identifiable neurological disease or other medical causation.^1,2^ FND accounts for a substantial proportion of neurology referrals,^3^ yet the condition remains widely misunderstood and treatment is often challenging to access.^4^ The impact of FND on daily functioning, independence and psychological well-being is profound, and consistently highlighted in patient experience and clinical research.^5^

Contemporary neurobiological models conceptualise FND as a disorder of disrupted integration between neural systems supporting emotional processing, bodily awareness (interoception) and cognitive control.^2,6–8^ Neuroimaging studies have demonstrated altered activation and microstructural changes in key regions within these networks, including the amygdala, insula and anterior cingulate cortex.^7,8^ A growing number of experimental studies show corresponding alterations in emotional responsivity and regulation,^8,9^ reduced interoceptive awareness and/or accuracy^10,11^ and difficulties with attentional control, conflict monitoring and other executive functions.^12,13^ Together, these findings suggest that disruptions in the coordination of visceral, affective and cognitive processes may interfere with the generation of coherent sensorimotor experiences, possibly contributing to the emergence and maintenance of functional symptoms.^2,7,8^

Clinically, individuals with FND describe symptoms that are variable, episodic and difficult to anticipate, with changes in motor control, sensory experiences or dissociative states occurring suddenly or shifting in intensity across contexts.^5,14^ Autonomic nervous system dysregulation has been recognised as a potential mechanism underpinning this variability.^8,15–17^ Many individuals with FND experience prominent autonomic and somatic symptoms such as palpitations, gastrointestinal discomfort, dizziness and other bodily sensations linked to emotional and arousal states.^15–17^ Recent research further suggests that affective and interoceptive challenges can modulate both subjective functional symptoms and autonomic responding in FND, supporting a role for dynamic interactions among emotional arousal, bodily signals and symptom expression.^16,17^

There is a developing evidence base for specialist treatments such as physiotherapy and cognitive–behavioural therapy;^18,19^ however, such treatments are often inaccessible to many with FND due to geographic, clinical or resource constraints.^20^ There is a need for alternatives that are feasible, acceptable and accessible and that might help to address the interoceptive, emotional and autonomic disturbances that are associated with FND.^8,10,11^

Somatic yoga is a potentially promising intervention due to its direct targeting of several neurobiological processes implicated in FND.^21^ It incorporates slow, accessible movement, breathwork, mindful sensory and interoceptive attention and gentle, body-based practices designed to modulate arousal and enhance sensorimotor coherence. Through these combined elements, somatic yoga aims to strengthen both bottom-up and top-down regulation of bodily and emotional states.^21,22^

Slow, deliberate movement and sustained sensory attention can increase activation within interoceptive networks, including the insula, supporting more accurate detection of bodily signals.^23^ Breath-focused practices enhance parasympathetic regulation and reduce sympathetic arousal,^22,24^ and yoga-based interventions have been associated with strengthened prefrontal–limbic connectivity and improvements in emotion regulation and stress responsivity.^25,26^ Beyond mechanistic pathways, yoga has been linked to benefits regarding fatigue, pain and quality of life across neurological and chronic health conditions,^27,28^ including conditions with mechanistic overlap with FND such as irritable bowel syndrome^29^ and fibromyalgia.^30^

To date, only two uncontrolled case series, by Kipnis et al,^31,32^ have examined individualised yoga-based interventions for people with functional (dissociative) seizures, reporting preliminary improvements in symptom management and well-being. A pilot study combining yoga with transcranial direct current stimulation (tDCS) has also reported reductions in dissociation and seizure frequency in functional seizures.^33^ Although these initial findings are encouraging, no controlled study has yet evaluated a structured yoga intervention for the broader FND population, preventing definitive conclusions regarding feasibility, acceptability or mechanistic relevance.^34^ Larger studies are needed in a wider range of FND phenotypes, including control groups, and with standardised intervention content.

This feasibility randomised controlled trial evaluated the delivery, acceptability and practical implementation of a 6-week somatic yoga intervention compared with an active, music-based relaxation programme for adults with FND. The primary objective was to assess feasibility and acceptability through recruitment, retention, adherence, adverse events and participant-reported experience. Secondary, exploratory aims were to examine preliminary changes in relevant outcome domains, including self-reported FND symptom burden, psychological symptoms (anxiety, depression, dissociation), quality of life, emotional well-being, interoceptive awareness and autonomic symptoms. A mixed-methods design, integrating quantitative outcomes with qualitative reflections, provided complementary insights into acceptability, perceived mechanisms and participant experiences.

The primary feasibility objectives were to assess whether (a) it would be possible to recruit the target sample size within the specified time frame; (b) retention would be ≥70% across the intervention period; (c) session attendance would be ≥70% on average; and (d) the intervention would be deemed acceptable to participants.

## Method

### Study design and participants

This single-site, parallel-arm, feasibility randomised controlled trial compared a 6-week, individually delivered somatic yoga intervention with a 6-week, music-based relaxation programme. The study was conducted in the Neurological Affective and Dissociative Symptoms Laboratory, Institute of Psychiatry, Psychology & Neuroscience (IoPPN), King’s College London. All participants provided written informed consent before enrolment. A patient and public involvement focus group, comprising individuals with lived experience of FND, was consulted during study development.

The authors assert that all procedures contributing to this work comply with the ethical standards of the relevant national and institutional committees on human experimentation, and with the Helsinki Declaration of 1975 as revised in 2013. All procedures involving human subjects/patients were approved by the King’s College London Health Faculties High Risk Research Ethics Committee (ref. no. HR/DP-24/25-46075). The trial was prospectively registered (no. ISRCTN73085690) in February 2025.

We aimed to enrol 30 participants in the study, with 15 to be randomised to the yoga intervention and 15 to the music relaxation control condition. The target sample size was deemed appropriate because the primary aims of the study were to assess feasibility rather than to test efficacy.^35^

Participants were eligible if they were ≥18 years old, fluent in English, had normal or corrected vision and a clinician-confirmed diagnosis of FND (motor, sensory or seizure symptoms). Exclusion criteria were major cardiovascular, psychiatric or neurological disorders likely to confound outcomes (e.g. heart disease, active psychosis, severe substance use disorder, epilepsy, multiple sclerosis); physical limitations preventing participation (e.g. severe tremor, limb paralysis, seizure frequency >10 per day); functional cognitive or sensory symptoms only; and concurrent engagement in body- or music-based therapeutic interventions that could not be paused during the study.

Recruitment and screening were conducted via self-referral through two pathways: (a) online advertisements posted on FND Hope UK, relevant Facebook and Instagram groups and King’s College London research volunteer pages; and (b) direct invitations sent to individuals listed in a pre-existing research registry held by the senior author (S.P.).

### Randomisation and blinding

The first author (E.K.-B.) delivered the somatic yoga intervention and was therefore not blind to group allocation. The second author (L.S.M.M.), who was fully blinded throughout data processing and statistical analysis, conducted all quantitative analyses, under the supervision of a statistician with expertise in clinical trials (J.H.).

Participants were randomised 1:1 to the yoga or music arm using a computer-generated allocation sequence created with the National Cancer Institute Clinical Trial Randomization Tool (https://ctrandomization.cancer.gov) and administered by S.P., who was not involved in the intervention delivery or outcome assessments. Allocation was automated and concealed until assignment, ensuring that group allocation could not be influenced by the researcher or therapist. All participants, regardless of group assignment, attended their first and last intervention sessions in person at IoPPN. The initial session served to introduce the yoga or music programme and build rapport, and the final in-person session provided an opportunity to integrate and consolidate the intervention, and to elicit participant feedback regarding their experience of the intervention and study procedures. All remaining sessions were delivered online, once per week, over 6 consecutive weeks.

### Interventions

Table 1 presents details of the somatic yoga and music relaxation control programmes. Detailed intervention manuals for the yoga and music arms, together with home practice logs, are available in the supplementary materials.

**Table 1:** Comparison of intervention parameters

Parameter	Somatic yoga	Music relaxation
Number of sessions	Six individual therapist-led sessions	6 weeks of self-guided practice
Session duration	60 min	30–60 min
Delivery format	Individual, therapist-led; initial and final sessions in-person at KCL; remaining sessions online	Self-guided; no therapist-led sessions
Facilitator	Somatic yoga therapist (E.K.-B.)	None (self-guided)
Home practice frequency	Recommended three to five times per week	Recommended three to five times per week
Home practice structure	Audiovisually guided practice provided from week 2 to improve accessibility and reduce cognitive load	Choice of three curated instrumental playlists per session
Practice log	Digital practice log, web-based	Digital practice log, web-based
Weekly check-ins	Yes – weekly online contact to monitor well-being and facilitate engagement	Yes – matched to yoga arm for contact and structure
Intervention components	Slow, accessible movement; breath-based regulation; interoceptive and sensory–motor awareness; visual and vestibular drills; grounding and balance tasks; restorative postures	Instrumental music listening; no movement, breathwork or interoceptive guidance included
Individualisation	Yes – content, pace and emphasis adapted by therapist to each participant’s reported experience within each session	No – standardised programme; choice of playlist only
Materials provided	Digital manual; practice log; audiovisually guided practice; instrumental playlist	Digital manual; practice log; three curated playlists

### Somatic yoga

Participants assigned to the yoga group received six 60 min individual online sessions with a somatic yoga therapist (E.K.-B.). Sessions integrated slow, accessible movement, breath-based regulation, interoceptive and sensory–motor awareness practices and restorative postures, selected for their established or emerging scientific rationale. Movement components emphasised attention-rich, novel motor exploration consistent with evidence for neuroplastic remapping and improved sensorimotor organisation.^36,37^ Breath practices (e.g. diaphragmatic and paced breathing) were included to support parasympathetic activation and autonomic regulation.^21,26,38^ Interoceptive and sensory-awareness exercises drew on validated frameworks of body awareness and insula-mediated emotional regulation.^39,40^

Visual and vestibular drills, gentle cranial nerve-oriented movements (e.g. eye-tracking) and grounding or balance tasks were incorporated to support sensory–motor integration, vestibulo-ocular function and autonomic regulation.^41–43^ Restorative postures and guided rest (e.g. Savasana) were included to consolidate practice effects and promote down-regulation.^24^ Participants received access to a digital manual, practice log and playlist matched to the music group. Home practice was recommended three to five times per week, with an audiovisual guided practice added after feedback in week 2 to improve accessibility and reduce cognitive load. Weekly online check-ins were used to monitor well-being and facilitate engagement. The somatic yoga intervention is a bottom-up, body-based approach that works through sensory and somatic experience rather than verbal or cognitive processing, and is not cognitively demanding. Sessions were paced entirely to the individual, and adapted by the therapist in response to each participant’s reported experience within each session. Participants with mixed FND presentations, including some functional cognitive symptoms, were therefore able to participate fully.

### Music relaxation

Participants assigned to the music group completed a 6-week programme of 30–60 min of self-guided instrumental music listening, three to five times per week. Participants were able to choose between three curated playlists for each session, including the yoga instrumental soundtrack. Weekly check-ins matched those of the yoga group for contact and structure. Participants received access to a secure digital folder containing a brief manual, three playlists (also used in the yoga group to maintain auditory equivalence) and a practice log. The practice log prompted participants to record the date and duration of each listening session, the playlist used and brief reflections on their experience (e.g. comfort, ease of attention, mood or sensory responses). These logs were used to assess adherence and to capture qualitative information about participants’ engagement with the listening practices. No movement, breathwork or interoceptive guidance was included in this condition, to ensure differentiation from the somatic yoga intervention.

The music-based control condition was selected to account for non-specific therapeutic factors such as guided attention, expectancy and regular contact with a practitioner. Music with slow tempo, harmonic simplicity and minimal rhythmic variation has been shown to promote parasympathetic activation, reduce physiological and psychological stress and engage neural circuits involved in emotional and interoceptive processing.^44–46^ By matching therapist contact time and providing a structured relaxation experience without incorporating bodily movement or interoceptive training, this control condition was designed to isolate the specific contribution of embodied components of the somatic yoga intervention.^47^

### Outcome measures

#### Feasibility outcomes

Feasibility was assessed through recruitment, retention, attendance and adherence rates. Acceptability was evaluated using participant feedback obtained during weekly check-ins and at the end-of-programme interview. Safety and procedural integrity were assessed through monitoring of adverse events and protocol deviations. Adverse events were defined as any unfavourable medical or psychological occurrence during participation (e.g. FND symptom flares, increased pain, fatigue or marked distress). Protocol deviations were defined as departures from the approved protocol, such as missed or rescheduled assessments, substantial changes to planned session delivery or eligibility issues identified following enrolment.

#### Secondary outcomes

Secondary outcome measures were administered at baseline, week 3, week 6 and 3-month follow-up (Table 2). The selected measures assessed recommended outcome domains including self-reported FND symptom burden, psychological symptoms (anxiety, depression, dissociation), quality of life and work/social functioning,^48^ in addition to domains of relevance to this particular study, specifically emotional functioning, interoceptive awareness and autonomic symptoms.^48^ A measure of lifetime adverse events (Traumatic Experiences Checklist, TEC^49^) and somatoform dissociation (Somatoform Dissociation Questionnaire, SDQ-20^50^) were also included at baseline. All measures demonstrated acceptable internal consistency in prior validation studies.

**Table 2:** Self-report secondary outcome measures

Measure	Construct assessed	Subscales	Administration schedule	Psychometric properties
Toronto Alexithymia Scale–20^51^	Difficulty in recognising and describing emotions	Difficulty identifying feelings; difficulty describing feelings; externally oriented thinking	Baseline, week 3, week 6, follow-up	20 items; Cronbach’s *α* = 0.73–0.80 across subscales; good test–retest reliability (*r* = 0.77); strong convergent and discriminant validity
Multidimensional Dissociation Inventory^52^	Pathological dissociation	Disengagement; depersonalisation; de-realisation; emotional constriction; memory disturbance; identity dissociation	Baseline, week 3, week 6, follow-up	30 items; *α* = 0.85–0.95 across subscales; high internal consistency and validity across clinical populations
Generalized Anxiety Disorder–7^53^	Generalised anxiety symptoms	Total score	Baseline, week 3, week 6, follow-up	7 items; *α* ≈ 0.92; good test–retest reliability (*r* = 0.83); validated against structured clinical interviews
Patient Health Questionnaire–8^54^	Depressive symptoms	Total score	Baseline, week 3, week 6, follow-up	8 items; *α* ≈ 0.86–0.88; strong construct validity; sensitive to clinical change
Work and Social Adjustment Scale^55^	Impairments in general functioning	Total score	Baseline, week 3, week 6, follow-up	5 items; *α* = 0.70–0.94; strong concurrent validity with disability and symptom measures
Patient Health Questionnaire–15^56^	Physical symptom severity	Total score	Baseline, week 3, week 6, follow-up	15 items; *α* ≈ 0.80; good construct validity and sensitivity in somatic populations
36-Item Short Form Health Survey^57^	Health-related quality of life	Physical functioning; role physical; role emotional; vitality; emotional well-being; social functioning; pain; general health	Baseline, week 3, week 6, follow-up	36 items; subscale *α* ≈ 0.78–0.93; widely validated across chronic health populations
Multidimensional Assessment of Interoceptive Awareness-2^58^	Interoceptive sensibility	Noticing; not-distracting; not-worrying; attention regulation; emotional awareness; self-regulation; body listening; trusting	Baseline, week 3, week 6, follow-up	32 items; *α* ≈ 0.66–0.87 across subscales; good construct validity
Body Perception Questionnaire– Short Form^59^	Body awareness and autonomic reactivity	Body awareness; autonomic reactivity composite	Baseline, week 3, week 6, follow-up	46 items; *α* ≈ 0.80–0.90 across subscales; strong structural validity; validated in clinical and general populations
Somatoform Dissociation Questionnaire-20 (SDQ-20^50^	Somatoform dissociation	Total score	Baseline	20 items; good internal consistency and test–retest reliability; widely used measure of somatoform dissociation in clinical samples
Traumatic Experiences Checklist (TEC^49^)	Exposure to potentially traumatic life events and perceived impact	Total events; event impact	Baseline	Validated self-report checklist with demonstrated reliability and validity in psychiatric and trauma-exposed populations
Functional Neurological Symptom Questionnaire (FNSQ^60^)	FND symptom burden	Symptom count; severity; impact	Baseline, week 3, week 6, follow-Up	28 items; unvalidated measure
Clinical Global Impression-Improvement (CGI-I^61^)	Self-reported global clinical change	Global improvement rating	Baseline, week 3, week 6, follow-up	Single-item clinician/self-report scale; widely used as outcome measure in clinical trials

KCL, King’s College London; FND, functional neurological disorder.

Cronbach’s *α* and psychometric properties drawn from validation studies cited.

### Data analysis

Descriptive statistics for feasibility data, including recruitment, adherence/retention, withdrawal, adverse events and acceptability, were calculated and reported. Demographic and clinical characteristics were presented for each group separately. Descriptive data (*n*, %) on the nature of reported FND symptoms were calculated and reported for both participant groups.

Quantitative analyses were conducted using R for macOS (version 4.5.1, R Foundation for Statistical Computing, Vienna, Austria; https://www.R-project.org/) and RStudio for macOS (Posit Software, PBC, Boston, MA, USA; https://www.posit.co/). Descriptive data (*M*, standard deviation) for all secondary outcome measures were calculated and reported by arm and time point (Supplementary Table 1). Linear mixed-effects models, with group (yoga intervention, music control), time (week 3, week 6, follow-up) and baseline outcome scores (baseline scores on included outcome measures) as fixed effects and participant as random effects, were run using the packages lme4 (Bates et al, 2015; https://cran.r-project.org/package=lme4) and emmeans (Lenth et al, 2025; https://CRAN.R-project.org/package=emmeans) on the following outcome measures: Functional Neurological Symptom Questionnaire^60^, Toronto Alexithymia Scale–20 (TAS-20)^51^, Multidimensional Dissociation Inventory (MDI)^52^, Patient Health Questionnaire–8 (PHQ-8)^54^, Patient Health Questionnaire–15 (PHQ-15)^56^, Generalized Anxiety Disorder–7^53^, Multidimensional Assessment of Interoceptive Awareness (MAIA)^58^, Body Perception Questionnaire–Short Form (BPQ)^59^, Short Form Health Survey-36^57^, Work and Social Adjustment Scale^55^ and Clinical Global Impression–Improvement (CGI-I)^61^. The yoga group and week 3 were the reference levels within these analyses. Effect sizes (contrast from each mixed-model/baseline standard deviation of music control group, Glass’s Δ) were calculated. Rates of missing data are presented for each outcome variable across the four time points (baseline, week 3, week 6, follow-up) in Supplementary Table 1.

Qualitative data, including practice logs and interviews, were analysed using reflexive thematic analysis.^62^ Coding focused on themes relating to intervention experience (yoga and music), perceived barriers and facilitators to feasibility and acceptability, and participant recommendations for refining the intervention and study procedures. Extended qualitative feedback, including participant reflections and session-end comments, is provided in Supplementary Table 4. These data were not available for all randomised participants, because these outcomes relied primarily on completion of home practice logs. Several participants reported difficulties using the digital logging system, resulting in incomplete or missing log data despite continued engagement with the intervention. Where available, qualitative data from end-of-study sessions supplemented log data; however, primary outcome analyses were restricted to participants with sufficient primary outcome data. This is reflected in the Consolidated Standards of Reporting Trials (CONSORT) flow diagram.

## Results

### Sample

Recruitment commenced in February 2025, with all 3-month follow-ups completed by September 2025. Baseline characteristics of participants who began the study and attended at least the first in-person intervention session are presented in Table 3. Mean body mass index was slightly greater in the yoga group, and more participants in the yoga group were currently single. A greater number of participants in the yoga group were also undergoing other current treatment (58%, *n* = 7) compared with the music group (9%, *n* = 1); because body-based therapies were an explicit exclusion criterion, these concurrent treatments comprised non-body-based therapies for FND (e.g. psychological therapies) and treatments for comorbid conditions unrelated to FND (details provided in Table 3). Primary FND symptoms were comparable between the two groups, with similar levels of bodily dissociation (SDQ-20) being reported. The groups endorsed the same average number of previous adverse life events (TEC) and indicated a comparable impact of these events.

**Table 3:** Demographic and clinical characteristics of participants

Variables	Yoga *N* = 12 *n* (%)	Music *N* = 11 *n* (%)
Age, years, *M* (s.d.)	45.9 (13.0)	44.9 (11.6)
Body mass index, *M* (s.d.)	29.7 (7.4)	26.0 (5.2)
Gender (% female)	11 (92)	11 (100)
Handedness (% right)	12 (100)	10 (91)
Smoking status (% yes)	3 (25)	0 (0)
Relationship (% single)	7 (58)	2 (18)
Dependents (% yes)	5 (42)	6 (55)
Education		
Compulsory	0 (0)	0 (0)
A-levels	5 (42)	4 (36)
Undergraduate or postgraduate	6 (50)	7 (64)
Employment (% employed)	6 (50)	4 (36)
Ethnicity		
White	9 (75)	7 (64)
Black	1 (8)	2 (18)
Asian	1 (8)	0 (0)
Mixed	1 (8)	2 (18)
Knowledge of heart rate	6 (50)	4 (36)
Current yoga practice (% yes)	2 (17)	2 (18)
Current music relaxation/listening (% yes)	2 (17)	4 (36)
Current physical health diagnosis (% yes)	8 (67)	9 (82)
Current mental health diagnosis (% yes)	10 (83)	7 (64)
Current medication (% yes)	9 (75)	10 (91)
Current other treatment (% yes)	7 (58)	1 (9)
Psychological	5 (71)	1 (100)
Other	2 (29)	0 (0)
Clinical Global Impression – severity, *M* (s.d.)	4.1 (0.9)	3.6 (1.0)
Primary self-reported FND symptoms		
Seizures	4 (33)	5 (45)
Tremors	3 (25)	2 (18)
Mobility/gait difficulties	3 (25)	5 (45)
Weakness/paralysis	4 (33)	4 (36)
Sensory symptoms	3 (25)	2 (18)
Jerks/spasms/tics	5 (42)	3 (27)
Speech difficulties	2 (17)	2 (18)
Somatoform Dissociation Questionnaire,^a^ *M* (s.d.)	34.25 (7.70)	29.36 (9.70)
Traumatic Experiences Checklist–Total Events,^a^ *M* (s.d.)	10.00 (5.10)	10.00 (7.22)
Traumatic Experiences Checklist–Event Impact,^a^ *M* (s.d.)	37.50 (17.53)	40.45 (30.43)

*M*, mean; FND, functional neurological disorder.

a. Measured at baseline.

### Feasibility measures

#### Recruitment

**Fig. 1:**
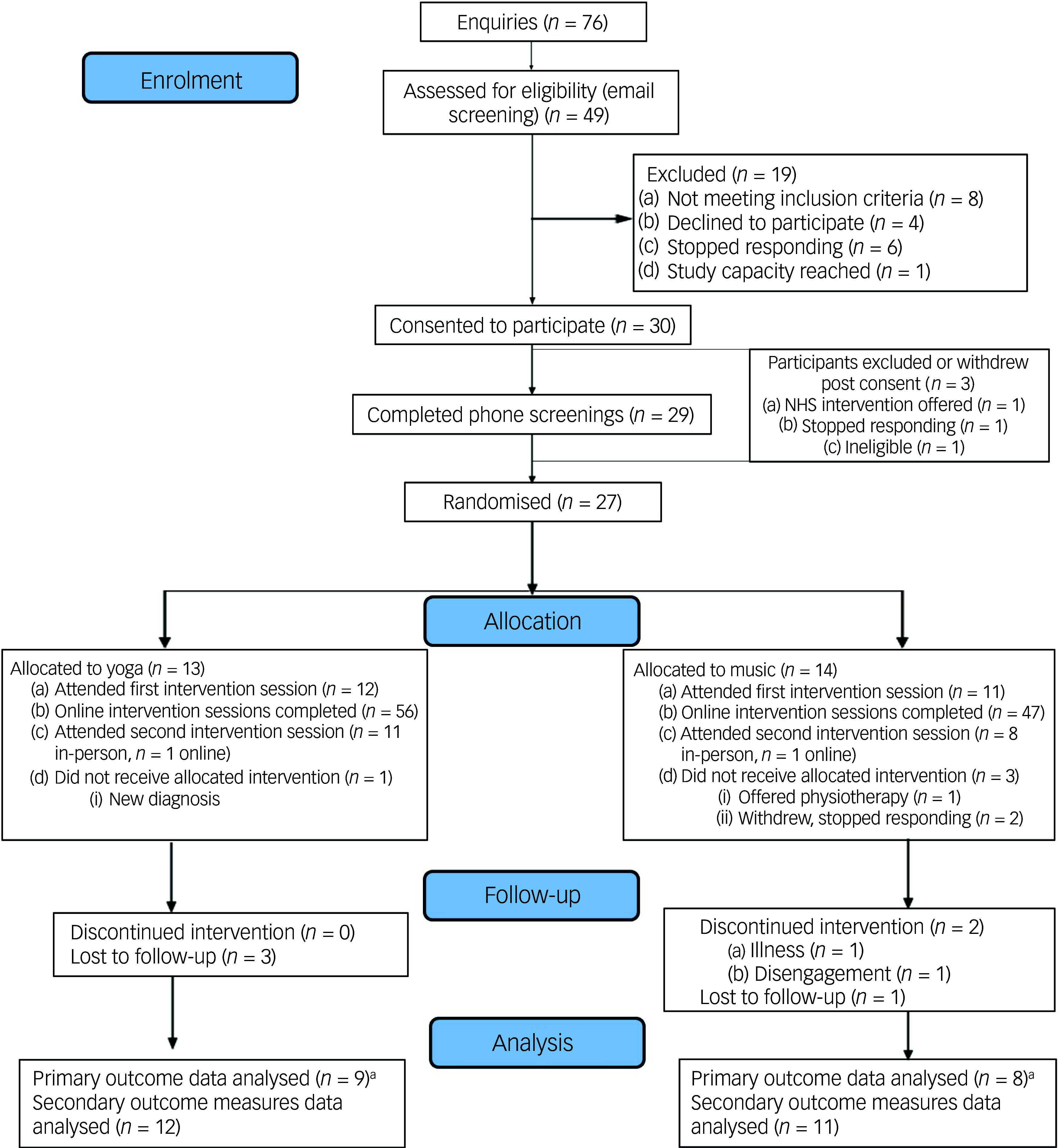
Consolidated Standards of Reporting Trials (CONSORT) flow diagram. NHS, National Health Service. a. Primary outcome analyses included only participants with sufficient practice log data. Some participants continued intervention participation but did not complete digital logs.

In total, 76 enquiries were received and 49 potential participants were screened. Twenty-seven individuals who enquired did not proceed to preliminary screening due to either declining, living outside of the UK, discontinuation of response or study capacity being reached. The CONSORT flow diagram illustrates participant progression through the trial (Fig. 1). Of those screened, 30 participants (representing 100% of the target sample size) provided informed consent and were enrolled in the study by 30 June 2025. Three participants (10%) withdrew (National Health Service intervention offered), stopped responding or were found to be ineligible (physical activity leading to functional neurological symptoms (FNS)) prior to randomisation, resulting in 27 (90%) proceeding to random group allocation (yoga, *n* = 13; music, *n* = 14). Four additional participants (13.3%) withdrew following randomisation but prior to the start of the intervention (symptom flare, new diagnosis, physical health issues), resulting in 23 participants (76.6%) taking part in the trial (yoga, *n* = 12; music, *n* = 11). Of the 27 randomised participants, 24 (89%) were recruited via the pre-existing research registry (yoga, *n* = 11, 85%, music: *n* = 13, 93%) and 3 (11%) via online advertisements (yoga, *n* = 2, 15%; music, *n* = 1, 7%). It should be noted that the majority of registry participants had not previously taken part in a study; most had enquired about prior studies but had been unable to participate for various reasons.

### Adherence/retention

During the intervention, two further participants (6.6%) from the music group disengaged and could not be reached for follow-up, resulting in 21 participants (70% of the initially consenting sample) completing the full intervention period (yoga, *n* = 12, music, *n* = 9). Twelve participants in the yoga group and 11 in the music group attended the first in-person session. On average, the yoga group attended 79.2% of online sessions across the 6-week study period (4.75 out of 6.0) and the music group attended 72.7% (4.36 out of 6.0; Supplementary Table 2). Eleven participants in the yoga group and eight in the music group attended the second, final, in-person session, alongside one participant from each group attending a version of this session online. Seventy-five per cent of the yoga group and 91% of the music group completed the 3-month follow-up activities.

### Withdrawal

Twenty-three per cent of consented participants (7 out of 30) withdrew prior to study completion, either post consent, post randomisation or during the trial.

### Adverse events

No intervention-related adverse events were observed during study participation. Five participants experienced either FND-related symptom flares (i.e. seizures, increased pain, fatigue, dizziness), medical complications or logistical challenges that affected attendance and adherence. However, these were consistent with the expected variability of the condition and were not considered intervention related. All five of these participants continued to participate in the trial; no intervention-related adverse events led to discontinuation of either intervention.

### Acceptability

Qualitative feedback supported the acceptability of the intervention. Most participants described positive or neutral experiences and perceived benefit, particularly within the yoga group. Barriers to engagement primarily involved usability of digital practice logs, with alternative communication (video call or email contact) sustaining overall participation. Difficulties with the web-based home practice logging system were documented for eight participants in the yoga arm (67%) and five in the music arm (45%); however, because logging difficulties were not systematically recorded as a binary variable, these figures reflect documented instances rather than a formal count. Of these participants, three in the yoga arm (25%) and two in the music arm (18%) reported home practice verbally during scheduled check-in calls as an alternative to digital logging. Beyond logged comments, feedback shared in the final sessions echoed these themes, with participants highlighting benefits during symptom flare-ups, use at stressful times and a desire to continue beyond the trial. Participant feedback on feasibility and acceptability is summarised in Supplementary Table 3.

### Secondary outcome measures

**Fig. 2:**
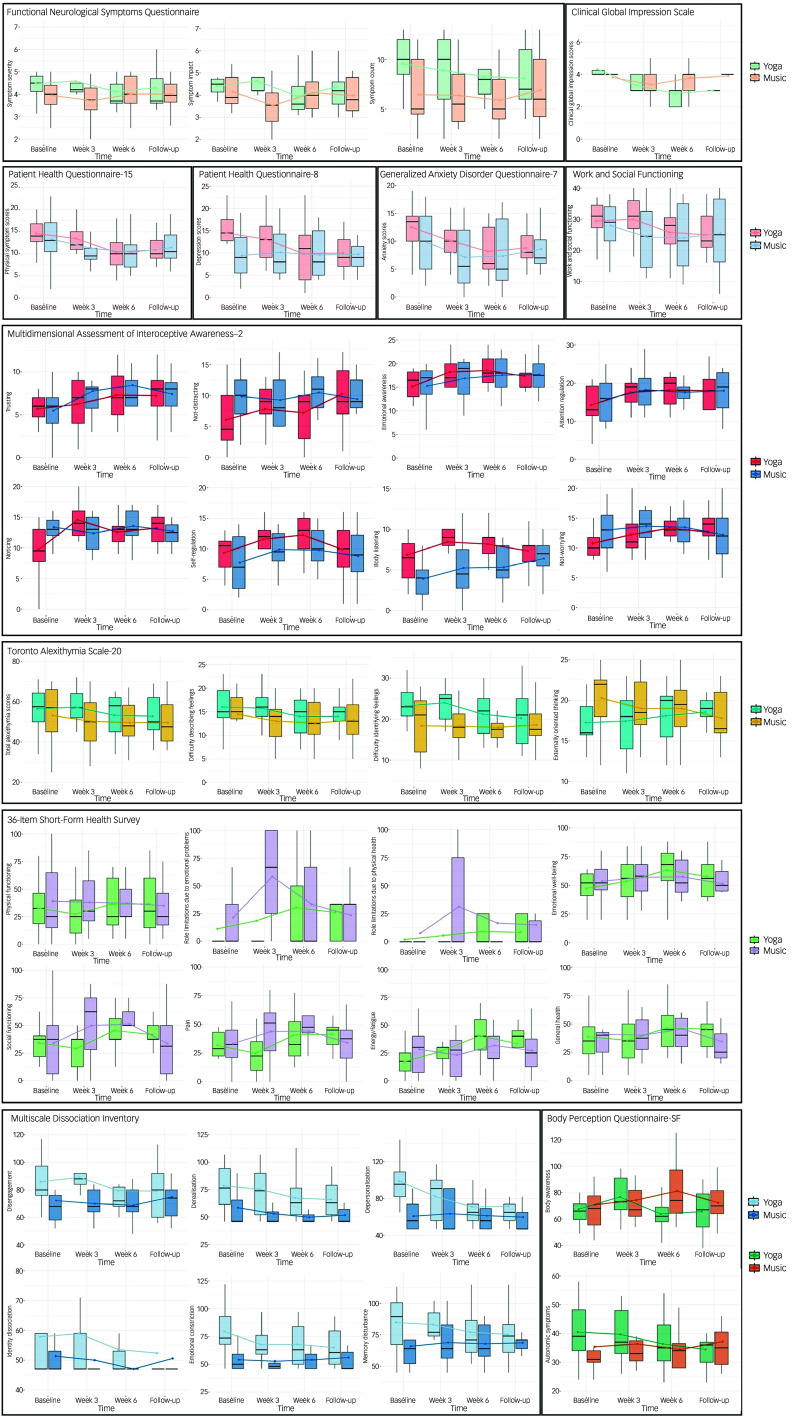
Outcome measures by group over time.

Statistical values for the linear mixed-effects models assessing secondary outcome variables are presented in Supplementary Table 5, with estimated intervention effects at each time point presented in Table 4 and Fig. 2. Given the sample size and aims of the study, the following will focus on effect size rather than statistical significance. Only medium and large standardised effect sizes (Δ ≥ 0.50) are discussed, given their potential practical or clinical relevance.

**Table 4:** Estimated intervention effects at each time point (linear mixed-model-estimated marginal means contrasts)

Variables	Contrast	Time point	Estimate	95% CI	Glass’s Δ
FNSQ severity	Yoga–Music	Week 3	0.30	−0.37, 0.96	0.37
	Yoga–Music	Week 6	−0.27	−0.90, 0.36	0.34
	Yoga–Music	Follow-up	−0.15	−0.80, 0.49	0.19
FNSQ impact	**Yoga–Music**	**Week 3**	**0.53**	**−0.18, 1.24**	**0.72**
	Yoga–Music	Week 6	−0.36	−1.05, 0.32	0.49
	Yoga–Music	Follow-up	0.05	−0.64, 0.75	0.07
FNSQ count	Yoga–Music	Week 3	−0.27	−2.76, 2.22	0.08
	Yoga–Music	Week 6	0.15	−2.24, 2.54	0.04
	Yoga–Music	Follow-up	−0.70	−3.11, 1.70	0.21
PHQ-8	Yoga–Music	Week 3	−0.58	−5.19, 4.03	0.11
	**Yoga–Music**	**Week 6**	**−3.15**	**−7.59, 1.29**	**0.58**
	Yoga–Music	Follow-up	−2.61	−7.01, 1.79	0.48
PHQ-15	Yoga–Music	Week 3	0.85	−1.88, 3.58	0.14
	Yoga–Music	Week 6	−0.91	−3.56, 1.74	0.15
	Yoga–Music	Follow-up	−1.15	−3.81, 1.52	0.19
GAD-7	Yoga–Music	Week 3	1.16	−2.92, 5.24	0.21
	Yoga–Music	Week 6	−0.87	−4.74, 3.00	0.16
	Yoga–Music	Follow-up	−1.59	−5.49, 2.30	0.28
TAS-20 total	Yoga–Music	Week 3	2.52	−5.03, 10.08	0.17
	Yoga–Music	Week 6	−0.13	−7.46, 7.20	0.01
	Yoga–Music	Follow-up	1.68	−5.55, 8.92	0.11
TAS-20 DDF	Yoga–Music	Week 3	1.52	−1.65, 4.68	0.29
	Yoga–Music	Week 6	−0.23	−3.31, 2.86	0.04
	Yoga–Music	Follow-up	0.59	−2.47, 3.64	0.11
TAS-20 DIF	Yoga–Music	Week 3	1.29	−2.94, 5.53	0.18
	Yoga–Music	Week 6	−1.25	−5.42, 2.92	0.17
	Yoga–Music	Follow-up	−1.16	−5.23, 2.92	0.16
TAS-20 EOT	Yoga–Music	Week 3	−0.13	−2.97, 2.71	0.03
	Yoga–Music	Week 6	1.61	−1.23, 4.45	0.42
	**Yoga–Music**	**Follow-up**	**2.60**	**−0.20, 5.40**	**0.68**
MDI D	Yoga–Music	Week 3	4.57	−7.67, 16.80	0.24
	Yoga–Music	Week 6	−2.54	−14.18, 9.10	0.13
	Yoga–Music	Follow-up	−1.41	−13.05, 10.20	0.07
MDI DP	Yoga–Music	Week 3	−0.71	−23.20, 21.76	0.02
	Yoga–Music	Week 6	−7.68	−29.80, 14.45	0.18
	Yoga–Music	Follow-up	−15.06	−36.70, 6.62	0.35
MDI DR	Yoga–Music	Week 3	2.63	−10.70, 15.97	0.13
	Yoga–Music	Week 6	−7.40	−20.60, 5.81	0.37
	Yoga–Music	Follow-up	−9.10	−22.10, 3.85	0.46
MDI MD	Yoga–Music	Week 3	4.30	−12.50, 21.08	0.13
	Yoga–Music	Week 6	−8.41	−24.50, 7.71	0.25
	Yoga–Music	Follow-up	−6.45	−22.60, 9.69	0.19
MDI EC	Yoga–Music	Week 3	0.77	−13.48, 15.00	0.03
	Yoga–Music	Week 6	−1.28	−15.27, 12.70	0.05
	Yoga–Music	Follow-up	7.77	−5.87, 21.40	0.32
MDI ID	Yoga–Music	Week 3	3.88	−6.73, 14.49	0.48
	Yoga–Music	Week 6	2.10	−7.89, 12.10	0.26
	Yoga–Music	Follow-up	−2.49	−12.66, 7.68	0.31
WSAS	Yoga–Music	Week 3	2.68	−3.00, 8.35	0.34
	Yoga–Music	Week 6	0.29	−5.17, 5.75	0.04
	Yoga–Music	Follow-up	0.00	−5.51, 5.51	0.00
CGI-I	**Yoga–Music**	**Week 3**	**−0.60**	**−1.49, 0.30**	**0.51**
	**Yoga–Music**	**Week 6**	**−1.42**	**−2.27, −0.57**	**1.21**
	**Yoga–Music**	**Follow-up**	**−1.19**	**−2.04, −0.34**	**1.02**
BPQ-SF BA	Yoga–Music	Week 3	3.59	−13.60, 20.77	0.15
	**Yoga–Music**	**Week 6**	**−16.20**	**−32.80, 0.41**	**0.70**
	Yoga–Music	Follow-up	−3.80	−20.40, 12.80	0.16
BPQ-SF ANS	Yoga–Music	Week 3	−0.19	−5.17, 4.79	0.01
	Yoga–Music	Week 6	−4.22	−9.04, 0.60	0.33
	Yoga–Music	Follow-up	−3.51	−8.20, 1.18	0.28
MAIA-2 N	**Yoga–Music**	**Week 3**	**3.01**	**0.42, 5.60**	**0.81**
	Yoga–Music	Week 6	−0.05	−2.50, 2.41	0.01
	Yoga–Music	Follow-up	1.30	−1.18, 3.79	0.35
MAIA-2 ND	Yoga–Music	Week 3	−0.23	−4.07, 3.60	0.05
	Yoga–Music	Week 6	−1.17	−4.92, 2.58	0.27
	Yoga–Music	Follow-up	1.99	−1.76, 5.74	0.46
MAIA-2 NW	Yoga–Music	Week 3	−0.35	−3.03, 2.34	0.08
	Yoga–Music	Week 6	1.63	−0.89, 4.15	0.39
	Yoga–Music	Follow-up	1.16	−1.37, 3.68	0.27
MAIA-2 AR	Yoga–Music	Week 3	0.25	−4.18, 4.68	0.04
	Yoga–Music	Week 6	1.89	−2.36, 6.14	0.31
	Yoga–Music	Follow-up	1.00	−3.30, 5.31	0.17
MAIA-2 EA	Yoga–Music	Week 3	1.98	−1.54, 5.49	0.44
	Yoga–Music	Week 6	1.52	−1.81, 4.85	0.34
	Yoga–Music	Follow-up	0.17	−3.22, 3.56	0.04
MAIA-2 SR	Yoga–Music	Week 3	1.31	−1.30, 3.92	0.27
	Yoga–Music	Week 6	2.04	−0.43, 4.51	0.43
	Yoga–Music	Follow-up	0.61	−1.90, 3.12	0.13
MAIA-2 BL	**Yoga–Music**	**Week 3**	**3.40**	**0.71, 6.10**	**1.28**
	**Yoga–Music**	**Week 6**	**2.83**	**0.22, 5.44**	**1.06**
	Yoga–Music	Follow-up	1.17	−1.45, 3.79	0.44
MAIA-2 T	Yoga–Music	Week 3	0.31	−2.33, 2.94	0.11
	Yoga–Music	Week 6	−0.33	−2.86, 2.19	0.11
	Yoga–Music	Follow-up	0.13	−2.41, 2.68	0.05
SF-36 PF	Yoga–Music	Week 3	−3.04	−12.14, 6.06	0.09
	Yoga–Music	Week 6	1.01	−7.62, 9.63	0.03
	Yoga–Music	Follow-up	2.79	−5.96, 11.55	0.08
SF-36 RLPH	**Yoga–Music**	**Week 3**	**−23.67**	**−48.30, 0.93**	**1.00**
	Yoga–Music	Week 6	−4.40	−27.10, 18.31	0.19
	Yoga–Music	Follow-up	−4.23	−27.20, 18.72	0.19
SF-36 RLEP	**Yoga–Music**	**Week 3**	**−28.57**	**−64.50, 7.41**	**0.84**
	Yoga–Music	Week 6	1.62	−31.10, 34.39	0.05
	Yoga–Music	Follow-up	9.86	−23.90, 43.65	0.29
SF-36 E/F	**Yoga–Music**	**Week 3**	**11.66**	**−2.90, 26.20**	**0.50**
	**Yoga–Music**	**Week 6**	**18.19**	**3.98, 32.40**	**0.79**
	**Yoga–Music**	**Follow-up**	**14.40**	**0.23, 28.60**	**0.62**
SF-36 EW	Yoga–Music	Week 3	−1.47	−17.50, 14.60	0.08
	**Yoga–Music**	**Week 6**	**9.05**	**−6.03, 24.10**	**0.51**
	**Yoga–Music**	**Follow-up**	**9.43**	**−5.87, 24.70**	**0.54**
SF-36 SF	**Yoga–Music**	**Week 3**	**−17.39**	**−36.70, 1.88**	**0.58**
	Yoga–Music	Week 6	−4.83	−23.10, 13.41	0.16
	Yoga–Music	Follow-up	7.69	−10.90, 26.25	0.25
SF-36 P	**Yoga–Music**	**Week 3**	**−13.43**	**−31.46, 4.59**	**0.69**
	Yoga–Music	Week 6	−3.85	−21.09, 13.39	0.20
	**Yoga–Music**	**Follow-up**	**9.81**	**−7.64, 27.27**	**0.50**
SF-36 GH	Yoga–Music	Week 3	−4.01	−15.28, 7.25	0.16
	Yoga–Music	Week 6	2.36	−8.32, 13.03	0.10
	Yoga–Music	Follow-up	4.96	−5.86, 15.78	0.20

FNSQ, Functional Neurological Symptom Questionnaire; PHQ-8, Patient Health Questionnaire–8; PHQ-15, Patient Health Questionnaire–15; GAD-7, Generalized Anxiety Disorder–7; TAS-20, Toronto Alexithymia Scale–20 (DDF, Difficulty Describing Feelings; DIF, Difficulty Identifying Feelings; EOT, Externally Oriented Thinking); MDI, Multidimensional Dissociation Inventory (D, Detachment; DP, Depersonalisation; DR, De-realisation; MD, Memory Disturbance; EC, Emotional Constriction; ID, Identity Dissociation); WSAS, Work and Social Adjustment Scale; CGI-I, Clinical Global Impression-Improvement; BPQ-SF, Body Perception Questionnaire–Short Form (ANS, Autonomic Nervous System; BA, Body Awareness); MAIA-2, Multidimensional Assessment of Interoceptive Awareness (N, Noticing; ND, Not Distracting; NW, Not Worrying; AR, Attention Regulation; EA, Emotional Awareness; SR, Self Regulation; BL, Body Listening; T, Total); SF-36, Short-Form Health Survey–36 (PF, Physical Functioning; RLPH, Role Limitations Physical Health; RLEP, Role Limitations Emotional Problems; E/F, Energy/Fatigue; EW, Emotional Well-being; SF, Social Functioning; P, Pain; GH, General Health).

Bold values indicate Glass’s Δ ≥ 0.50 (medium or large effect size).

Medium effect sizes were observed across multiple outcomes. At week 3, the self-reported impact of FND symptoms was elevated in the music group (Δ = 0.72), whereas pain (Short-Form Health Survey (SF-36 P), Δ = 0.69) was reduced and symptom improvement was greater relative to the study screening interview (Clinical Global Impression–Improvement (CGI-I), Δ = 0.51) in the yoga group. By contrast, elevated identity dissociation (Multidimensional Dissociation Inventory (MDI ID), Δ = 0.48) was seen at this time point in the yoga group. At week 6, depression (Patient Health Questionnaire–8 (PHQ-8), Δ = 0.58) and body awareness (Body Perception Questionnaire (BPQ BA), Δ = 0.70) scores were lower and energy/fatigue scores were higher (indicative of more energy and less fatigue; SF-36 E/F, Δ = 0.79) in the yoga group. At 3-month follow-up, elevated energy was again reported in the yoga group (Δ = 0.62), as well as more externally oriented thinking (Toronto Alexithymia Scale–20 (TAS-20 EOT), Δ = 0.68) and reduced de-realisation (MDI DR, Δ = 0.46).

Large effect size also emerged for a handful of outcomes. Elevated noticing (Multidimensional Assessment of Interoceptive Awareness version 2 (MAIA-2 N), Δ = 0.81) and body listening (MAIA-2 BL, Δ = 1.28) were seen in the yoga group relative to the music control at week 3. However, higher scores on role limitations due to physical health (SF-36 RLPH, Δ = 1.00) and emotional problems (SF-36 RLEP, Δ = 0.84), indicating self-reported improvements in these domains, were seen in the music group relative to yoga at week 3. At week 6, body listening remained elevated in the yoga group (MAIA-2 BL, Δ = 1.06), as well as greater self-reported symptom improvement relative to study entrance (lower CGI-I scores, Δ = 1.21). This finding further remained at 3-month follow-up (CGI-I, Δ = 1.02). All of the above results point towards meaningful group differences across these outcome measures.

## Discussion

This study is the first randomised feasibility trial of a somatic yoga intervention for adults living with FND. The trial demonstrated that a 6-week, individually delivered, somatic yoga programme is both feasible and acceptable, and revealed preliminary, hypothesis-generating signals of therapeutic potential, particularly in self-reported FND symptom improvement and interoceptive awareness.

### Feasibility and acceptability

Recruitment targets were met in full, with strong retention overall. Completion rates differed by arm, with notably higher retention in the yoga group, and adherence was generally high across both arms. These findings reinforce the feasibility and acceptability of both interventions. Importantly, no intervention-related adverse events were observed. Whereas some participants experienced symptom flares, health complications or attendance barriers, these reflected the expected variability of FND rather than intervention harms, reinforcing the acceptability and safety of hybrid-delivered, embodied therapeutic practices in this population.

These outcomes are broadly consistent with prior feasibility trials in FND, where fluctuating symptoms and comorbidities have been noted to limit retention. For instance, psychological interventions such as eye movement desensitisation and reprocessing (MODIFI^63^) and cognitive–behavioural therapy (CODES^18^) for FND have reported follow-up completion rates of between 68 and 85%,^18,63^ whereas physiotherapy-based feasibility trials have often achieved higher retention.^64,65^ Against this background, our overall completion rate of 70%, alongside 100% retention in the yoga arm, supports the feasibility and acceptability of hybrid-delivered yoga practice for this population. The absence of attrition in the yoga arm is particularly notable, suggesting strong acceptability and engagement. Qualitative feedback reinforced this, with participants describing the sessions as calming, supportive and relevant for daily coping. A majority of participants, however, reported that the digital logging system was difficult to use. This was not only burdensome for those with neurodivergent traits but also presented wider accessibility challenges across the cohort. Importantly, these difficulties did not prevent engagement: participants adapted through alternative contact (direct therapist communication, telephone feedback), sustaining overall participation. Inclusion of patients in the co-design of digital logging or outcome-monitoring tools will be critical in future studies.

The adaptability of the intervention was further illustrated by continued engagement during periods of significant life stress (e.g. bereavement, moving house, holidays). This flexibility highlights the acceptability of the intervention under real-world conditions, where disruptions and competing demands are common, and illustrates the strength of online delivery in maintaining engagement. The initial and final in-person sessions are also likely to have contributed to therapeutic alliance and participant engagement throughout the programme.

Although the web-based home practice logging system presented usability challenges for some participants, this did not affect engagement with the intervention itself, and alternative methods of contact were available to both groups equally. The primary limitation this presents is one of data capture rather than therapeutic delivery, and would be straightforwardly addressed in future iterations through a mobile logging app.

### Preliminary trends in secondary outcomes

Large effect sizes highlight self-reported global clinical improvement and interoception-related outcomes as potentially promising signals. At week 3, marked increases in MAIA-2 ‘Noticing’ and ‘Body Listening’ were seen in the yoga group, whereas the music group reported reduced role limitations due to physical and emotional problems. Self-reported FND symptom improvement (CGI-I) showed large and sustained effects from week 6 through to 3-month follow-up, and ‘Body Listening’ remained elevated at week 6 in the yoga group. Taken together, these findings suggest that somatic yoga may support changes in self-reported overall symptom improvement and interoceptive sensibility, and both interventions may confer short-term benefits in other specific symptom domains. These findings also extend previous reports of the impact of yoga on interoception, autonomic regulation and broader aspects of well-being.^21,25,26^

Medium effect sizes were also observed across multiple secondary outcomes, with patterns varying by domain and time point. Early in the intervention (week 3), a mixed profile emerged. The music control group reported reduced pain (SF-36 Pain). In contrast, the yoga intervention was associated with greater self-reported global clinical improvement relative to study screening (CGI-I), alongside early increases in interoceptive sensibility, suggesting engagement of broader regulatory processes beyond immediate symptom modulation.^26,58^ At the same time, elevated identity-related dissociation was observed in the yoga group at week 3. This was an unexpected result that could be reflective of heightened self-attention during early embodied practice, possibly generating some dissociative symptoms.

By week 6, the pattern more consistently favoured the yoga intervention across well-being-related outcomes, including reduced depressive symptoms (PHQ-8), reduced body awareness difficulties (BPQ Body Awareness) and increased vitality (SF-36 Energy/Fatigue), alongside sustained improvements in interoceptive sensibility. At 3-month follow-up, increased energy was again reported in the yoga group, together with changes in emotional processing and dissociation including reduced de-realisation and increased externally oriented thinking. Although both study arms showed improvement on selected outcomes, effects in the music control condition were more limited and time-specific, including early reductions in pain and symptom perception, consistent with short-term calming effects. In contrast, the yoga intervention was associated with larger, more sustained benefits, particularly for FND symptom improvement and aspects of interoceptive sensibility. Evaluating yoga against an active control therefore supports the interpretation that embodied, movement-based components may confer added value beyond general relaxation alone.

These secondary outcomes should be interpreted as exploratory, and used to inform outcome measure selection and mechanistic hypotheses for a fully powered trial.

### Strengths and limitations

This study had several strengths. Although the use of yoga has been investigated in depression, anxiety and chronic pain, studies in FND have thus far been limited to small case series in functional seizures,^31,32,34^ with one pilot study combining yoga with tDCS.^33^ The present trial therefore represents the first randomised evaluation of yoga in a broader FND population. The randomised design and use of an active comparator reduced expectancy and attention biases. Mixed-methods analysis provided both quantitative outcome data and qualitative depth, enabling a richer understanding of feasibility and potential mechanisms. The intervention was designed and delivered in a participant-centred manner, meaning that participants were given choice and agency in their practice, sessions emphasised safety and regulation over performance and pacing was responsive to individual capacity. This approach may have helped to maintain engagement even during periods of stress such as bereavement, relocation or holidays, underscoring the accessibility and resilience of the model.

Several limitations warrant consideration. The dual role of the researcher and therapist may have introduced bias, although the study was conducted under academic supervision. In addition, the lack of blinding of participants to treatment allocation may have influenced responses on the self-reported outcome measures. These risks were partly mitigated by adherence to the core pre-specified study protocol with only minor adaptations (e.g. addition of a video/audio guide to support home practice), and by independent blinded administration of outcome assessments (Y.B.) and analysis of feasibility, demographic and laboratory-based data (L.S.M.M.).

Digital logging challenges highlighted accessibility barriers that may have influenced adherence. The recruitment pathway included individuals from a pre-existing research registry held by the senior author, which may have introduced familiarity bias. Participants with prior research experience may have been more comfortable with study procedures, although it should be noted that only a small number of these participants had completed a study – most had enquired about research opportunities but had not enrolled in a study for a variety of reasons. Future trials should monitor and report the proportion of registry versus community recruitment by arm.

A greater proportion of yoga group participants were receiving other current treatments at baseline (58%) compared with the music control group (9%). Because these were non-body-based therapies, direct interference with the intervention is unlikely; however, the imbalance represents a potential confound for secondary outcome interpretation and should be noted as a limitation.

Medication changes were not systematically recorded as part of the study protocol. To the best of our knowledge, only one participant underwent a medication change during the trial and the practical impact on outcomes is therefore likely to be minimal. Nevertheless, future trials should systematically record medication changes at every outcome assessment time point.

Recruitment was conducted through non-clinical pathways, which may increase the potential for misdiagnosis relative to samples being recruited through tertiary clinical services. Although all participants provided clinician-confirmed diagnoses, the level of specialist oversight varied.

Finally, the intervention period of 6 weeks, although appropriate for feasibility testing, may not capture the full potential of yoga-based interventions in a chronic and fluctuating condition such as FND.

This feasibility trial demonstrates that somatic yoga is safe and acceptable for adults with FND, with promising signals of benefit in self-reported FND symptom improvement and interoceptive sensibility. By directly targeting emotional processing, interoceptive awareness and autonomic regulation, yoga may help to address mechanistic domains thought to be important in FND yet not directly targeted by current treatments.^18,19^ Although secondary outcomes must be regarded as exploratory trends within a small sample, our findings provide support for a fully powered randomised controlled trial.

## Supporting information

10.1192/bjo.2026.12003.sm001 Kennedy-Barnes et al. supplementary material Kennedy-Barnes et al. supplementary material

## Data Availability

The anonymised data-set will be made available in response to reasonable request to the corresponding author (S.P.), on a case-by-case basis. Completion of a Data Sharing Agreement will be required, facilitated by the King’s College London Data Management team.

## Supplementary material

The supplementary material is available online at https://doi.org/10.1192/bjo.2026.12003
