# Supplementary material for: Somatic yoga therapy for functional neurological disorder: feasibility randomised controlled trial
Source: BJPsych Open. 2026 Jun 2;12(4):e151. doi: 10.1192/bjo.2026.12003 (PMC13237120; doi:10.1192/bjo.2026.12003)
Supplement: Kennedy-Barnes et al. supplementary material [file S2056472426120031sup001.docx]

**Supplementary Materials**

**Table S1.** Outcome measures by group across time.

|  | **Yoga** | | | | **Music** | | | |
| --- | --- | --- | --- | --- | --- | --- | --- | --- |
|  | **Baseline**  **N=12** | **Week 3**  **N=9** | **Week 6**  **N=11** | **Follow-up**  **N=9** | **Baseline**  **N=11** | **Week 3**  **N=8** | **Week 6**  **N=9** | **Follow-up**  **N=10** |
| **FNSQ count** | 9.58 (2.54) | 8.89 (3.82) | 8.27 (2.76) | 8.11 (3.14) | 6.46 (3.39) | 6.38 (3.34) | 5.89 (3.22) | 6.90 (3.64) |
| **FNSQ severity** | 4.47 (.82) | 4.59 (.83) | 4.11 (.80) | 4.29 (1.18) | 3.96 (.79) | 3.71 (.96) | 4.01 (.86) | 4.00 (.71) |
| **FNSQ impact** | 4.42 (.83) | 4.64 (.87) | 3.96 (.89) | 4.38 (1.18) | 4.16 (.74) | 3.54 (1.13) | 4.11 (.95) | 3.99 (.82) |
| **TAS-20** | 56.50 (10.90) | 57.20 (8.41) | 53.3 (10.4) | 52.80 (12.1) | 53.10 (14.70) | 50.20 (14.3) | 49.6 (12.6)* | 49.60 (11.2) |
| **TAS-20 Difficulty Describing** | 16.00 (4.69) | 15.80 (4.15) | 14.00 (4.22) | 14.00 (4.22) | 14.50 (5.26) | 13.00 (4.81) | 12.60 (5.48)* | 13.20 (5.29) |
| **TAS-20 Difficulty Identifying** | 23.20 (5.96) | 24.00 (4.95) | 21.20 (5.49) | 20.20 (7.89) | 18.40 (7.22) | 18.20 (5.95) | 18.00 (4.24)* | 18.60 (5.50) |
| **TAS-20 Externally Oriented Thinking** | 17.20 (2.96) | 17.40 (3.84) | 18.10 (3.65) | 18.60 (3.58) | 20.30 (3.80) | 19.00 (4.21) | 19.00 (4.00)* | 17.80 (3.64) |
| **MDI Disengagement** | 85.90 (16.00) | 89.10 (14.50) | 79.50 (18.10) | 83.80 (23.20) | 72.20 (19.10) | 70.00 (10.70) | 68.90 (13.10) | 74.90 (19.30) |
| **MDI Depersonalisation** | 111.00 (36.10) | 92.00 (37.80) | 80.00 (38.00) | 71.70 (29.80) | 79.00 (43.50) | 63.50 (22.80) | 61.40 (16.90)* | 70.80 (39.30) |
| **MDI Derealisation** | 78.40 (21.60) | 75.60 (22.60) | 67.40 (20.20) | 65.90 (18.40) | 58.50 (20.00) | 53.00 (12.10) | 49.80 (6.30) | 59.40 (25.40) |
| **MDI Memory Disturbance** | 92.60 (27.00) | 91.70 (20.30) | 77.10 (21.70) | 81.90 (29.50) | 78.70 (33.40) | 68.90 (19.00) | 67.90 (16.60) | 78.40 (32.50) |
| **MDI Emotion Constriction** | 79.50 (22.60) | 67.80 (14.50) | 67.90 (18.80) | 72.30 (27.50) | 60.70 (24.00) | 52.80 (13.00) | 54.00 (8.34) | 56.10 (17.60) |
| **MDI Identity Dissociation** | 57.80 (20.40) | 58.90 (23.70) | 53.50 (12.40) | 52.30 (12.20) | 51.40 (8.09) | 50.00 (8.48) | 47.00 (0.00) | 50.60 (8.10) |
| **PHQ-8** | 14.50 (5.16) | 13.10 (5.16) | 9.82 (6.72) | 10.00 (4.09) | 9.46 (5.43) | 10.10 (6.20) | 9.56 (5.59) | 9.70 (4.72) |
| **PHQ-15** | 14.60 (3.68) | 13.40 (3.50) | 10.40 (3.93) | 10.90 (3.14) | 13.30 (5.95) | 11.20 (5.39) | 10.30 (4.30) | 11.40 (4.14) |
| **GAD-7** | 12.50 (4.30) | 10.10 (3.92) | 8.18 (4.79) | 8.78 (3.93) | 9.82 (5.60) | 7.12 (6.18) | 7.33 (6.46) | 8.60 (4.33) |
| **MAIA Noticing** | 10.50 (5.14) | 14.60 (3.00) | 12.50 (2.30) | 13.20 (2.68) | 13.40 (3.70) | 12.40 (3.07) | 13.60 (3.21) | 12.70 (2.54) |
| **MAIA Not Distracting** | 6.08 (4.72) | 7.78 (4.26) | 7.18 (5.12) | 10.20 (5.19) | 9.82 (4.31) | 9.25 (4.59) | 10.40 (3.36) | 9.40 (4.30) |
| **MAIA Not Worrying** | 10.80 (4.09) | 12.20 (3.90) | 13.30 (3.93) | 12.70 (4.58) | 13.00 (4.22) | 13.60 (3.20) | 13.40 (3.13) | 12.20 (4.37) |
| **MAIA Attention Regulation** | 14.20 (5.34) | 17.60 (4.69) | 18.20 (4.26) | 18.00 (5.00) | 15.80 (6.06) | 18.20 (5.85) | 17.60 (2.65) | 18.00 (5.54) |
| **MAIA Emotional Awareness** | 15.20 (4.22) | 18.20 (3.42) | 18.50 (3.24) | 17.30 (4.42) | 15.30 (4.47) | 16.90 (4.61) | 17.60 (4.25) | 17.70 (4.11) |
| **MAIA Self-Regulation** | 9.33 (3.08) | 11.60 (3.40) | 12.30 (3.41) | 9.89 (4.62) | 7.73 (4.78) | 9.88 (3.40) | 9.78 (3.67) | 8.80 (5.05) |
| **MAIA Body Listening** | 6.83 (3.76) | 8.44 (2.01) | 8.18 (2.09) | 7.33 (2.24) | 3.91 (2.66) | 5.25 (3.92) | 5.33 (2.65) | 6.40 (2.91) |
| **MAIA Trusting** | 5.67 (1.72) | 6.22 (3.35) | 7.27 (2.97) | 7.22 (3.90) | 5.46 (2.88) | 7.75 (3.54) | 8.44 (3.00) | 7.40 (4.12) |
| **BPQ Body Awareness** | 67.50 (13.60) | 76.70 (16.30) | 63.80 (13.70) | 65.60 (17.80) | 70.80 (23.30) | 74.20 (20.70) | 81.20 (24.00)* | 72.30 (15.70) |
| **BPQ Autonomic Symptoms** | 40.50 (11.60) | 39.70 (8.85) | 36.40 (9.56) | 34.40 (8.17) | 35.40 (12.70) | 36.40 (11.10) | 34.40 (7.11)* | 37.20 (11.40) |
| **WSAS** | 29.40 (6.95) | 30.00 (7.55) | 25.80 (8.02) | 25.60 (7.83) | 27.90 (7.83) | 24.20 (10.60) | 24.00 (10.80) | 25.50 (12.10) |
| **SF-36 Physical Functioning** | 32.90 (23.20) | 27.20 (21.50) | 36.40 (24.90) | 37.20 (30.70) | 39.10 (33.50) | 38.10 (27.90) | 37.20 (28.00) | 35.00 (28.60) |
| **SF-36 Role Limitations due to Physical Health** | 2.08 (7.22) | 5.56 (11.00) | 9.09 (12.60) | 8.33 (12.50) | 7.50 (23.70) | 31.20 (43.80) | 16.70 (35.40) | 15.00 (31.60) |
| **SF-36 Role Limitations due to Emotional Problems** | 11.10 (29.60) | 18.50 (37.70) | 30.30 (40.70) | 25.90 (32.40) | 21.20 (34.20) | 58.30 (46.30) | 33.30 (44.10) | 23.30 (22.50) |
| **SF-36 Energy/Fatigue** | 17.10 (13.20) | 26.70 (16.00) | 40.50 (18.80) | 34.40 (17.00) | 28.20 (23.10) | 23.10 (19.30) | 31.70 (23.00) | 27.00 (21.80) |
| **SF-36 Emotional Wellbeing** | 47.30 (15.50) | 53.30 (22.30) | 63.30 (22.70) | 57.30 (17.60) | 53.50 (17.60) | 57.00 (19.80) | 57.30 (16.60) | 51.20 (15.90) |
| **SF-36 Social Functioning** | 34.40 (17.80) | 29.20 (19.80) | 45.50 (19.60) | 41.70 (16.50) | 34.10 (30.20) | 50.00 (34.70) | 51.40 (17.10) | 33.80 (29.50) |
| **SF-36 Pain** | 31.70 (11.10) | 24.70 (17.70) | 40.70 (22.50) | 41.10 (13.40) | 33.20 (19.50) | 43.80 (27.90) | 44.20 (16.30) | 34.00 (20.90) |
| **SF-36 General Health** | 38.80 (24.70) | 35.60 (23.20) | 46.40 (19.90) | 43.30 (26.20) | 40.90 (24.80) | 40.00 (18.90) | 45.60 (23.50) | 34.50 (23.10) |
| **CGI-I** | 4.33 (.65) | 3.33 (.50) | 2.82 (.75) | 3.00 (.50) | 3.82 (1.17) | 3.38 (.92) | 3.78 (.97) | 3.90 (1.60) |

*Notes*.*n=8

**Table S2.** Feasibility metrics: home practice completion, adherence rates across study arms.

| **Feasibility metric** | **Yoga** | **Music** | **Combined total** |
| --- | --- | --- | --- |
| Home practice log completion (%) | 78.2 | 77.3 | 77.8 |
| High adherence (≥80% sessions) | 7/12 | 7/11 | 14/23 |
| Moderate adherence (50–79% sessions) | 3/12 | 2/11 | 5/23 |
| Low adherence (<50% sessions) | 2/12 | 2/11 | 4/23 |
| Common reasons for dropout/low adherence | Illness, overload | Tech issues, disengagement | — |

**Table S3.** Qualitative acceptability indicators*.*

| **Domain** | **Illustrative quotes from participant logs and feedback** |
| --- | --- |
| **Attendance** | “The sessions gave me something to look forward to each week.” *(Yoga participant)*  “I felt calmer and more connected with my body after practice.” *(Yoga participant)*  “Loved feeling relaxed and the sounds of the rainforest … so calm, helps to settle before bed.” *(Music participant)* |
| **Adherence** | “Practices helped me feel more present and less worried.” *(Yoga participant)*  “More time to self … this is helping to learn about anxiety and taking control of it.” *(Music participant)* |
| **Barriers to engagement** | “The online log was tricky – I preferred telling the therapist directly.” *(Yoga participant)*  “Pain made it hard to keep up with logs, but I kept practicing.” *(Yoga participant)*  “Has found it hard logging with the online log format.” *(Music participant)* |
| **Acceptability (benefits & perceived value)** | “I looked forward to practice, even on bad days.” *(Yoga participant)*  “Feeling more able to cope with the day. A lot happier.” *(Yoga participant)*  “Relaxed. Calm. Fell asleep when listening.” *(Music participant)* |
| **Adaptability & inclusion** | “I valued the flexibility – being able to modify practices made it accessible.” *(Yoga participant)*  “Practices brought joy after having done them.” *(Yoga participant)*  “Relaxed. Refreshed. Mind still racing even after the full hour.” *(Music participant)* |

*Notes*. Feedback was provided by participants from both arms, though qualitative data were richer in the yoga group. Themes derived from participant interviews and practice logs using reflexive thematic analysis. This synthesis highlights the complex relationship between “objective” engagement metrics and participants’ lived experience.

**Table S4.** Participant feedback at study end and logged reflections.

| **Theme** | **Illustrative Feedback (Logs + Final Session)** | **Implications for Feasibility** |
| --- | --- | --- |
| **1. Benefits of Practice** | “I feel calmer and more positive after practice, even when my symptoms flare” (Yoga).  “This is no longer a chore — it brings joy after having done it” (Yoga).  “Music relaxation helped me sleep and feel less anxious, even if I struggled to stay focused” (Music). | Practices were perceived as meaningful and beneficial, enhancing relaxation, calmness, and positivity, supporting acceptability. |
| **2. Use During Symptoms and Stress** | Yoga participants described using practices *during seizure onset* or at times when symptoms would usually escalate, reporting reduced intensity afterwards.  “Breathwork was especially useful before bed and when I felt symptoms coming on” (Yoga).  Music participants sometimes used recordings when stressed: “It calmed me a bit, but the symptoms still came” (Music). | Yoga showed potential symptom-management value; music offered some stress relief but with mixed effects. Highlights feasibility of interventions being used flexibly in real- world contexts. |
| **3. Challenges with Digital Logging** | “I did the practices but couldn’t manage the log format” (Yoga).  “The log was awkward — I’d rather just say it in the call” (Music).  Final feedback: majority requested a *dedicated app* instead of web-based logging. | Digital logging created a barrier; future iterations should adopt app- based or simplified logging methods to improve adherence data capture. |
| **4. Practice Preferences** | Music group: “I often fell asleep — shorter tracks would be better.”  Yoga group: valued variety (breathwork, shaking, restorative poses), with flexibility to adapt practice length to symptoms. | Music practices may require adaptation (shorter sessions). Yoga practices were well- received with adaptability considered a strength. |
| **5. Continuation Beyond Study** | “I’d like to carry on — this is the only positive thing since my diagnosis” (Yoga).  “Even if I fell asleep sometimes, I would still like more of this” (Music).  Final session: several yoga participants requested to keep recordings, and some reported actively seeking local classes. | Strong participant demand to continue suggests high acceptability and sustainability potential. |

In addition to adherence data, participants provided qualitative feedback both through weekly practice logs and in the final in-person sessions. Their reflections centered on benefits of the practices, use during symptoms and stress, challenges with digital logging, preferences regarding practice format, and desire to continue beyond the study. These insights complement the quantitative feasibility outcomes by highlighting lived experience and practical considerations for future implementation.

**Table S5.** Linear mixed effects models on secondary outcome variables.

|  | ***ß*** | **SE** | **95% CI** | **t** | **p** |
| --- | --- | --- | --- | --- | --- |
| **FNSQ Severity** |  |  |  |  |  |
| *Intercept* | 1.260 | .810 | -3.28, 2.848 | 1.555 | .136 |
| *Baseline score* | .707 | .173 | .368, 1.046 | 4.087 | .001 |
| *Group: Group2* | -.296 | .324 | -.931, .339 | -.913 | .368 |
| *Group: Group2 x Time: FollowUp* | .448 | .275 | -.092, .988 | 1.627 | .114 |
| *Group: Group2 x Time: Week6* | .567 | .274 | .029, 1.104 | 2.065 | .047 |
| *Time: FollowUp* | -.276 | .194 | -.657, .105 | -1.419 | .166 |
| *Time: Week6* | -.324 | .191 | -.699, .051 | -1.695 | .100 |
| **FNSQ Impact** |  |  |  |  |  |
| *Intercept* | .967 | .913 | -.822, 2.757 | 1.060 | .302 |
| *Baseline score* | .778 | .197 | .392, 1.165 | 3.952 | .001 |
| *Group: Group2* | -.528 | .349 | -1.211, .156 | -1.513 | .140 |
| *Group: Group2 x Time: FollowUp* | .475 | .288 | -.090, 1.039 | 1.649 | .110 |
| *Group: Group2 x Time: Week6* | .892 | .287 | .329, 1.455 | 3.106 | .004 |
| *Time: FollowUp* | -.214 | .203 | -.612, .185 | -1.051 | .302 |
| *Time: Week6* | -.459 | .201 | -.852, -.066 | -2.287 | .029 |
| **FNSQ Count** |  |  |  |  |  |
| *Intercept* | .487 | 1.828 | -3.096, 4.070 | .266 | .793 |
| *Baseline score* | .821 | .172 | .485, 1.158 | 4.788 | .00 |
| *Group: Group2* | .270 | 1.216 | -2.113, 2.654 | .222 | .826 |
| *Group: Group2 x Time: FollowUp* | .433 | .953 | -1.435, 2.302 | .455 | .653 |
| *Group: Group2 x Time: Week6* | -.418 | .949 | -2.278, 1.442 | -.441 | .662 |
| *Time: FollowUp* | .042 | .673 | -1.277, 1.361 | .063 | .950 |
| *Time: Week6* | .282 | .663 | -1.016, 1.581 | .426 | .673 |
| **PHQ-8** |  |  |  |  |  |
| *Intercept* | 4.097 | 2.709 | -1.212, 9.405 | 1.512 | .143 |
| *Baseline score* | .591 | .159 | .279, .902 | 3.717 | .001 |
| *Group: Group2* | .580 | 2.264 | -3.857, 5.016 | .256 | .799 |
| *Group: Group2 x Time: FollowUp* | 2.029 | 2.167 | -2.218, 6.277 | .936 | .356 |
| *Group: Group2 x Time: Week6* | 2.573 | 2.153 | -1.646, 6.792 | 1.195 | .240 |
| *Time: FollowUp* | -2.382 | 1.529 | -5.379, .616 | -1.557 | .129 |
| *Time: Week6* | -2.781 | 1.490 | -5.701, .140 | -1.866 | .070 |
| **PHQ-15** |  |  |  |  |  |
| *Intercept* | 4.295 | 1.959 | .455, 8.134 | 2.192 | .041 |
| *Baseline score* | .586 | .120 | .351, .822 | 4.879 | .000 |
| *Group: Group2* | -.849 | 1.336 | -3.468, 1.769 | -.636 | .530 |
| *Group: Group2 x Time: FollowUp* | 1.997 | 1.055 | -.070, 4.064 | 1.894 | .068 |
| *Group: Group2 x Time: Week6* | 1.760 | 1.050 | -.299, 3.819 | 1.676 | .104 |
| *Time: FollowUp* | -1.723 | .745 | -3.182, -.263 | -2.313 | .028 |
| *Time: Week6* | -2.076 | .734 | -3.515, -.637 | -2.828 | .008 |
| **GAD-7** |  |  |  |  |  |
| *Intercept* | 2.705 | 2.360 | -1.921, 7.331 | 1.146 | .263 |
| *Baseline score* | .541 | .154 | .239, .843 | 3.512 | .002 |
| *Group: Group2* | -1.162 | 2.009 | -5.099, 2.776 | -.578 | .566 |
| *Group: Group2 x Time: FollowUp* | 2.755 | 2.043 | -1.249, 6.759 | 1.349 | .187 |
| *Group: Group2 x Time: Week6* | 2.028 | 2.029 | -1.948, 6.004 | 1.000 | .325 |
| *Time: FollowUp* | -.944 | 1.442 | -3.770, 1.882 | -.655 | .517 |
| *Time: Week6* | -1.221 | 1.404 | -3.974, 1.531 | -.870 | .390 |
| **TAS-20 Total** |  |  |  |  |  |
| *Intercept* | 19.748 | 7.175 | 5.684, 33.811 | 2.752 | .012 |
| *Baseline score* | .649 | .119 | .416, .882 | 5.466 | .000 |
| *Group: Group2* | -2.525 | 3.716 | -9.808, 4.758 | -.680 | .501 |
| *Group: Group2 x Time: FollowUp* | .841 | 3.604 | -6.223, 7.905 | .233 | .817 |
| *Group: Group2 x Time: Week6* | 2.657 | 3.657 | -4.511, 9.825 | .726 | .473 |
| *Time: FollowUp* | -2.010 | 2.542 | -6.992, 2.972 | -.791 | .435 |
| *Time: Week6* | -2.557 | 2.484 | -7.426, 2.312 | -1.029 | .311 |
| **TAS-20 DDF** |  |  |  |  |  |
| *Intercept* | 5.466 | 2.335 | .889, 10.043 | 2.341 | .030 |
| *Baseline score* | .651 | .131 | .393, .908 | 4.950 | .000 |
| *Group: Group2* | -1.517 | 1.552 | -4.558, 1.524 | -.978 | .335 |
| *Group: Group2 x Time: FollowUp* | .932 | 1.394 | -1.800, 3.664 | .668 | .509 |
| *Group: Group2 x Time: Week6* | 1.744 | 1.417 | -1.034, 4.522 | 1.230 | .228 |
| *Time: FollowUp* | -.788 | .984 | -2.716, 1.140 | -.801 | .430 |
| *Time: Week6* | -1.573 | .965 | -3.465, .318 | -1.631 | .113 |
| **TAS-20 DIF** |  |  |  |  |  |
| *Intercept* | 9.083 | 3.224 | 2.765, 15.402 | 2.818 | .010 |
| *Baseline score* | .631 | .127 | .383, .879 | 4.979 | .00 |
| *Group: Group2* | -1.294 | 2.074 | -5.360, 2.771 | -.624 | .537 |
| *Group: Group2 x Time: FollowUp* | 2.451 | 1.823 | -1.123, 6.025 | 1.344 | .189 |
| *Group: Group2 x Time: Week6* | 2.548 | 1.854 | -1.086, 6.182 | 1.374 | .179 |
| *Time: FollowUp* | -2.808 | 1.287 | -5.330, -.287 | -2.183 | .037 |
| *Time: Week6* | -2.455 | 1.261 | -4.926, .016 | -2.183 | .060 |
| **TAS-20 EOT** |  |  |  |  |  |
| *Intercept* | 4.521 | 2.983 | -1.325, 10.368 | 1.516 | .146 |
| *Baseline score* | .710 | .161 | .395, 1.025 | 4.419 | .00 |
| *Group: Group2* | .129 | 1.398 | -2.611, 2.869 | .092 | .927 |
| *Group: Group2 x Time: FollowUp* | -2.732 | 1.423 | -5.521, .056 | -1.921 | .065 |
| *Group: Group2 x Time: Week6* | -1.742 | 1.447 | -4.577, 1.093 | -1.204 | .238 |
| *Time: FollowUp* | 1.686 | 1.003 | -.279, 3.651 | 1.682 | .103 |
| *Time: Week6* | 1.538 | .981 | -.384, 3.461 | 1.568 | .127 |
| **MDI Disengagement** |  |  |  |  |  |
| *Intercept* | 23.089 | 11.80 | -.038, 46.217 | 1.957 | .064 |
| *Baseline score* | .735 | .128 | .483, .986 | 5.716 | .00 |
| *Group: Group2* | -4.574 | 6.021 | -16.375, 7.227 | -.760 | .452 |
| *Group: Group2 x Time: FollowUp* | 5.983 | 5.882 | -5.547, 17.512 | 1.017 | .317 |
| *Group: Group2 x Time: Week6* | 7.113 | 5.839 | -4.331, 18.557 | 1.218 | .232 |
| *Time: FollowUp* | -2.923 | 4.148 | -11.053, 5.207 | -.705 | .486 |
| *Time: Week6* | -6.409 | 4.045 | -14.336, 1.519 | -1.584 | .122 |
| **MDI Depersonalisation** |  |  |  |  |  |
| *Intercept* | 15.973 | 14.63 | -12.706, 44.653 | 1.092 | .287 |
| *Baseline score* | .683 | .117 | .454, .911 | 5.855 | .00 |
| *Group: Group2* | .712 | 11.01 | -20.860, 22.283 | .065 | .949 |
| *Group: Group2 x Time: FollowUp* | 14.345 | 9.189 | -3.664, 32.355 | 1.561 | .129 |
| *Group: Group2 x Time: Week6* | 6.969 | 9.347 | -11.351, 25.290 | .746 | .462 |
| *Time: FollowUp* | -16.339 | 6.482 | -29.043, -3.635 | -2.521 | .017 |
| *Time: Week6* | -10.608 | 6.373 | -23.098, 1.883 | -1.664 | .106 |
| **MDI Derealisation** |  |  |  |  |  |
| *Intercept* | 10.246 | 10.87 | -11.066, 31.557 | .942 | .357 |
| *Baseline score* | .854 | .131 | .597, 1.111 | 6.510 | .00 |
| *Group: Group2* | -2.631 | 6.533 | -15.434, 10.173 | -.403 | .690 |
| *Group: Group2 x Time: FollowUp* | 11.735 | 5.437 | 1.079, 22.390 | 2.158 | .039 |
| *Group: Group2 x Time: Week6* | 10.031 | 5.427 | -.607, 20.668 | 1.848 | .075 |
| *Time: FollowUp* | -11.021 | 3.839 | -18.546, -3.497 | -2.871 | .008 |
| *Time: Week6* | -12.576 | 3.789 | -20.003, -5.150 | -3.319 | .002 |
| **MDI Memory Disturbance** |  |  |  |  |  |
| *Intercept* | 26.039 | 11.89 | 2.735, 49.342 | 2.190 | .040 |
| *Baseline score* | .674 | .114 | .452, .897 | 5.933 | .00 |
| *Group: Group2* | -4.302 | 8.236 | -20.44, 11.841 | -.522 | .605 |
| *Group: Group2 x Time: FollowUp* | 10.755 | 7.347 | -3.644, 25.155 | 1.464 | .154 |
| *Group: Group2 x Time: Week6* | 12.715 | 7.301 | -1.595, 27.026 | 1.742 | .092 |
| *Time: FollowUp* | -7.713 | 5.180 | -17.866, 2.440 | -1.489 | .147 |
| *Time: Week6* | -12.266 | 5.082 | -22.226, -2.305 | -2.413 | .022 |
| **MDI Emotion Constriction** |  |  |  |  |  |
| *Intercept* | 24.625 | 10.88 | 3.308, 45.943 | 2.264 | .033 |
| *Baseline score* | .544 | .128 | .293, .795 | 4.250 | .00 |
| *Group: Group2* | -.771 | 6.994 | -14.480, 12.937 | -.110 | .913 |
| *Group: Group2 x Time: FollowUp* | -7.00 | 5.956 | -18.673, 4.673 | -1.175 | .249 |
| *Group: Group2 x Time: Week6* | 2.047 | 5.926 | -9.567, 13.661 | .345 | .732 |
| *Time: FollowUp* | 6.091 | 4.196 | -2.134, 14.316 | 1.452 | .156 |
| *Time: Week6* | -.434 | 4.131 | -8.530, 7.663 | -.105 | .917 |
| **MDI Identity Dissociation** |  |  |  |  |  |
| *Intercept* | 32.795 | 7.962 | 17.189, 48.401 | 4.119 | .00 |
| *Baseline score* | .428 | .121 | .190, .666 | 3.521 | .003 |
| *Group: Group2* | -3.879 | 5.225 | -14.119, 6.362 | -.742 | .462 |
| *Group: Group2 x Time: FollowUp* | 6.370 | 5.533 | -4.475, 17.215 | 1.151 | .258 |
| *Group: Group2 x Time: Week6* | 1.774 | 5.496 | -8.999, 12.546 | .323 | .749 |
| *Time: FollowUp* | -6.837 | 3.904 | -14.488, .814 | -1.751 | .089 |
| *Time: Week6* | -4.640 | 3.798 | -12.085, 2.804 | -1.222 | .230 |
| **WSAS** |  |  |  |  |  |
| *Intercept* | 2.000 | 5.051 | -7.899, 11.899 | .396 | .697 |
| *Baseline score* | .966 | .161 | .649, 1.282 | 5.985 | .00 |
| *Group: Group2* | -2.678 | 2.782 | -8.130, 2.774 | -.963 | .343 |
| *Group: Group2 x Time: FollowUp* | 2.678 | 2.413 | -2.052, 7.407 | 1.110 | .276 |
| *Group: Group2 x Time: Week6* | 2.387 | 2.402 | -2.322, 7.095 | .993 | .328 |
| *Time: FollowUp* | -3.920 | 1.704 | -7.259, -.581 | -2.301 | .028 |
| *Time: Week6* | -4.258 | 1.676 | -7.544, -.972 | -2.540 | .016 |
| **CGI-I** |  |  |  |  |  |
| *Intercept* | .040 | 1.215 | -2.340, 2.421 | .033 | .974 |
| *Baseline score* | .741 | .272 | .207, 1.275 | 2.718 | .013 |
| *Group: Group2* | .596 | .439 | -.264, 1.456 | 1.358 | .183 |
| *Group: Group2 x Time: FollowUp* | .594 | .415 | -.219, 1.408 | 1.432 | .162 |
| *Group: Group2 x Time: Week6* | .823 | .412 | .016, 1.631 | 1.998 | .055 |
| *Time: FollowUp* | -.366 | .292 | -.940, .207 | -1.253 | .220 |
| *Time: Week6* | -.454 | .286 | -1.015, .107 | -1.585 | .123 |
| **BPQ Body Awareness** |  |  |  |  |  |
| *Intercept* | 65.824 | 14.04 | 38.311, 93.338 | 4.689 | .00 |
| *Baseline score* | .154 | .192 | -.222, .530 | .801 | .432 |
| *Group: Group2* | -3.590 | 8.44 | -20.140, 12.961 | -.425 | .673 |
| *Group: Group2 x Time: FollowUp* | 7.391 | 7.936 | -8.164, 22.945 | .931 | .359 |
| *Group: Group2 x Time: Week6* | 19.785 | 8.076 | 3.957, 35.614 | 2.450 | .020 |
| *Time: FollowUp* | -8.590 | 5.604 | -19.574, 2.393 | -1.533 | .135 |
| *Time: Week6* | -11.759 | 5.485 | -22.509, -1.008 | -2.144 | .039 |
| **BPQ Autonomic Symptoms** |  |  |  |  |  |
| *Intercept* | 9.345 | 3.498 | 2.489, 16.201 | 2.672 | .012 |
| *Baseline score* | .737 | .076 | .589, .886 | 9.761 | .00 |
| *Group: Group2* | .190 | 2.456 | -4.623, 5.004 | .078 | .939 |
| *Group: Group2 x Time: FollowUp* | 3.322 | 2.877 | -2.316, 8.960 | 1.155 | .256 |
| *Group: Group2 x Time: Week6* | 4.031 | 2.907 | -1.666, 9.728 | 1.387 | .174 |
| *Time: FollowUp* | -2.353 | 2.032 | -6.336, 1.631 | -1.158 | .255 |
| *Time: Week6* | -1.650 | 1.959 | -5.489, 2.189 | -.842 | .405 |
| **MAIA-2 Noticing** |  |  |  |  |  |
| *Intercept* | 12.057 | 1.294 | 9.521, 14.593 | 9.319 | .00 |
| *Baseline score* | .243 | .092 | .063, .422 | 2.653 | .017 |
| *Group: Group2* | -3.007 | 1.279 | -5.513, -.500 | -2.351 | .023 |
| *Group: Group2 x Time: FollowUp* | 1.702 | 1.576 | -1.387, 4.791 | 1.080 | .288 |
| *Group: Group2 x Time: Week6* | 3.053 | 1.563 | -.011, 6.117 | 1.953 | .059 |
| *Time: FollowUp* | -1.427 | 1.111 | -3.604, .750 | -1.284 | .208 |
| *Time: Week6* | -2.040 | 1.068 | -4.134, .055 | -1.909 | .064 |
| **MAIA-2 Not-Distracting** |  |  |  |  |  |
| *Intercept* | 3.262 | 1.605 | .116, 6.408 | 2.032 | .053 |
| *Baseline score* | .579 | .173 | .240, .918 | 3.343 | .003 |
| *Group: Group2* | .233 | 1.868 | -3.428, 3.894 | .125 | .901 |
| *Group: Group2 x Time: FollowUp* | -2.226 | 1.347 | -4.866, .414 | -1.653 | .108 |
| *Group: Group2 x Time: Week6* | .938 | 1.342 | -1.693, 3.569 | .699 | .490 |
| *Time: FollowUp* | 2.283 | .951 | .419, 4.148 | 2.400 | .022 |
| *Time: Week6* | .492 | .940 | -1.349, 2.334 | .524 | .604 |
| **MAIA-2 Not-Worrying** |  |  |  |  |  |
| *Intercept* | 4.348 | 1.537 | 1.336, 7.361 | 2.829 | .008 |
| *Baseline score* | .681 | .109 | .467, .895 | 6.248 | .00 |
| *Group: Group2* | .345 | 1.324 | -2.250, 2.941 | .261 | .795 |
| *Group: Group2 x Time: FollowUp* | -1.505 | 1.623 | -4.685, 1.675 | -.928 | .360 |
| *Group: Group2 x Time: Week6* | -1.976 | 1.610 | -5.130, 1.179 | -1.227 | .228 |
| *Time: FollowUp* | .295 | 1.143 | -1.945, 2.536 | .258 | .798 |
| *Time: Week6* | 1.507 | 1.103 | -.654, 3.668 | 1.366 | .180 |
| **MAIA-2 Attention Regulation** |  |  |  |  |  |
| *Intercept* | 14.147 | 2.706 | 8.843, 19.451 | 5.228 | .00 |
| *Baseline score* | .258 | .162 | -.059, .576 | 1.594 | .128 |
| *Group: Group2* | -.252 | 2.174 | -4.512, 4.009 | -.116 | .908 |
| *Group: Group2 x Time: FollowUp* | -.752 | 1.964 | -4.601, 3.097 | -.383 | .704 |
| *Group: Group2 x Time: Week6* | -1.639 | 1.954 | -5.469, 2.191 | -.839 | .408 |
| *Time: FollowUp* | .591 | 1.386 | -2.126, 3.308 | .426 | .673 |
| *Time: Week6* | .685 | 1.360 | -1.981, 3.350 | .503 | .618 |
| **MAIA-2 Emotional Awareness** |  |  |  |  |  |
| *Intercept* | 11.970 | 2.885 | 6.315, 17.624 | 4.149 | .001 |
| *Baseline score* | .444 | .176 | .099, .790 | 2.522 | .022 |
| *Group: Group2* | -1.976 | 1.730 | -5.368, 1.415 | -1.142 | .260 |
| *Group: Group2 x Time: FollowUp* | 1.806 | 1.739 | -1.602, 5.214 | 1.038 | .307 |
| *Group: Group2 x Time: Week6* | .455 | 1.729 | -2.933, 3.843 | .263 | .849 |
| *Time: FollowUp* | -1.298 | 1.227 | -3.703, 1.107 | -1.058 | .298 |
| *Time: Week6* | -.229 | 1.199 | -2.578, 2.120 | -.191 | .849 |
| **MAIA-2 Self-Regulation** |  |  |  |  |  |
| *Intercept* | 5.250 | 1.431 | 2.446, 8.054 | 3.670 | .001 |
| *Baseline score* | .756 | .126 | .509, 1.002 | 6.004 | .00 |
| *Group: Group2* | -1.311 | 1.285 | -3.289, 1.207 | -1.021 | .314 |
| *Group: Group2 x Time: FollowUp* | .705 | 1.332 | -1.907, 3.316 | .529 | .601 |
| *Group: Group2 x Time: Week6* | -.733 | 1.324 | -3.327, 1.862 | -.554 | .584 |
| *Time: FollowUp* | -1.813 | .940 | -3.655, .030 | -1.928 | .062 |
| *Time: Week6* | -.019 | .917 | -1.817, 1.778 | -.021 | .983 |
| **MAIA-2 Body Listening** |  |  |  |  |  |
| *Intercept* | 8.971 | 1.369 | 6.288, 11.654 | 6.554 | .00 |
| *Baseline score* | -.038 | .158 | -.348, .271 | -.243 | .811 |
| *Group: Group2* | -3.401 | 1.323 | -5.994, -.808 | -2.571 | .014 |
| *Group: Group2 x Time: FollowUp* | 2.234 | 1.211 | -.139, 4.606 | 1.845 | .074 |
| *Group: Group2 x Time: Week6* | .575 | 1.205 | -1.786, 2.936 | .477 | .636 |
| *Time: FollowUp* | -1.242 | .855 | -2.919, .434 | -1.452 | .156 |
| *Time: Week6* | -.556 | .837 | -2.197, 1.084 | -.665 | .511 |
| **MAIA-2 Trusting** |  |  |  |  |  |
| *Intercept* | .240 | 1.818 | -3.322, 3.803 | .132 | .896 |
| *Baseline score* | 1.162 | .284 | .606, 1.718 | 4.095 | .001 |
| *Group: Group2* | -.307 | 1.292 | -2.840, 2.266 | -.238 | .813 |
| *Group: Group2 x Time: FollowUp* | .174 | 1.124 | -2.029, 2.377 | .155 | .878 |
| *Group: Group2 x Time: Week6* | .639 | 1.117 | -1.550, 2.827 | .572 | .571 |
| *Time: FollowUp* | .320 | .792 | -1.232, 1.872 | .404 | .689 |
| *Time: Week6* | .182 | .779 | -1.345, 1.708 | .233 | .817 |
| **SF-36 PF** |  |  |  |  |  |
| *Intercept* | .263 | 3.772 | -7.130, 7.656 | .070 | .945 |
| *Baseline score* | .939 | .070 | .802, 1.075 | 13.447 | .00 |
| *Group: Group2* | 3.040 | 4.477 | -5.734, 11.815 | .679 | .501 |
| *Group: Group2 x Time: FollowUp* | -5.834 | 4.493 | -14.640, 2.972 | -1.299 | .204 |
| *Group: Group2 x Time: Week6* | -4.049 | 4.466 | -12.802, 4.705 | -.907 | .372 |
| *Time: FollowUp* | 6.559 | 3.170 | .346, 12.771 | 2.069 | .047 |
| *Time: Week6* | 4.592 | 3.097 | -1.478, 10.663 | 1.483 | .148 |
| **SF-36 RLPH** |  |  |  |  |  |
| *Intercept* | 3.550 | 7.954 | -12.041, 19.140 | .446 | .658 |
| *Baseline score* | .711 | .222 | .275, 1.147 | 3.198 | .005 |
| *Group: Group2* | 23.665 | 12.11 | -.068, 47.399 | 1.954 | .057 |
| *Group: Group2 x Time: FollowUp* | -19.437 | 14.87 | -48.582, 9.708 | -1.307 | .200 |
| *Group: Group2 x Time: Week6* | -19.270 | 14.78 | -48.234, 9.693 | -1.304 | .201 |
| *Time: FollowUp* | 2.963 | 10.15 | -16.931, 22.858 | .292 | .772 |
| *Time: Week6* | 3.857 | 9.77 | -15.291, 23.005 | .395 | .695 |
| **SF-36 RLEP** |  |  |  |  |  |
| *Intercept* | 17.224 | 11.95 | -6.205, 40.652 | 1.441 | .156 |
| *Baseline score* | .461 | .191 | .087, .835 | 2.414 | .024 |
| *Group: Group2* | 28.565 | 17.75 | -6.214, 63.344 | 1.610 | .114 |
| *Group: Group2 x Time: FollowUp* | -38.427 | 21.99 | -81.524, 4.671 | -1.748 | .089 |
| *Group: Group2 x Time: Week6* | -30.189 | 21.85 | -73.008, 12.631 | -1.382 | .176 |
| *Time: FollowUp* | 6.740 | 15.49 | -23.626, 37.107 | .435 | .666 |
| *Time: Week6* | 7.212 | 14.95 | -22.095, 36.518 | .482 | .632 |
| **SF-36 E/F** |  |  |  |  |  |
| *Intercept* | 17.520 | 5.543 | 6.656, 28.384 | 3.161 | .004 |
| *Baseline score* | .692 | .165 | .368, 1.015 | 4.187 | .001 |
| *Group: Group2* | -11.664 | 7.119 | -25.616, 2.288 | -1.639 | .112 |
| *Group: Group2 x Time: FollowUp* | -2.738 | 5.678 | -13.866, 8.391 | -.482 | .633 |
| *Group: Group2 x Time: Week6* | -6.523 | 5.659 | -17.613, 4.568 | -1.153 | .258 |
| *Time: FollowUp* | 4.861 | 4.009 | -2.997, 12.719 | 1.212 | .235 |
| *Time: Week6* | 10.573 | 3.951 | 2.830, 18.317 | 2.676 | .012 |
| **SF-36 EW** |  |  |  |  |  |
| *Intercept* | 26.135 | 10.00 | 6.536, 45.735 | 2.614 | .015 |
| *Baseline score* | .591 | .176 | .246, .935 | 3.362 | .004 |
| *Group: Group2* | 1.471 | 7.902 | -14.017, 16.959 | .186 | .853 |
| *Group: Group2 x Time: FollowUp* | -10.898 | 8.809 | -28.163, 6.367 | -1.237 | .225 |
| *Group: Group2 x Time: Week6* | -10.524 | 8.748 | -27.669, 6.621 | -1.203 | .237 |
| *Time: FollowUp* | 3.544 | 6.214 | -8.635, 15.724 | .570 | .572 |
| *Time: Week6* | 8.989 | 6.030 | -2.830, 20.808 | 1.491 | .145 |
| **SF-36 SF** |  |  |  |  |  |
| *Intercept* | 13.582 | 8.613 | -3.299, 30.463 | 1.577 | .126 |
| *Baseline score* | .489 | .155 | .185, .794 | 3.152 | .006 |
| *Group: Group2* | 17.389 | 9.484 | -1.199, 35.977 | 1.833 | .075 |
| *Group: Group2 x Time: FollowUp* | -25.076 | 9.588 | -43.867, -6.285 | -2.615 | .014 |
| *Group: Group2 x Time: Week6* | -12.556 | 9.540 | -31.254, 6.143 | -1.316 | .198 |
| *Time: FollowUp* | 11.950 | 6.765 | -1.308, 25.209 | 1.767 | .088 |
| *Time: Week6* | 14.270 | 6.615 | 1.304, 27.236 | 2.157 | .039 |
| **SF-36 Pain** |  |  |  |  |  |
| *Intercept* | 11.299 | 9.744 | -7.799, 30.398 | 1.160 | .261 |
| *Baseline score* | .563 | .236 | .101, 1.025 | 2.390 | .033 |
| *Group: Group2* | 13.431 | 8.854 | -3.923, 30.784 | 1.517 | .140 |
| *Group: Group2 x Time: FollowUp* | -23.244 | 8.227 | -39.370, -7.119 | -2.825 | .009 |
| *Group: Group2 x Time: Week6* | -9.579 | 8.184 | -25.619, 6.460 | -1.171 | .252 |
| *Time: FollowUp* | 14.499 | 5.806 | 3.119, 25.879 | 2.497 | .019 |
| *Time: Week6* | 9.761 | 5.693 | -1.398, 20.919 | 1.714 | .097 |
| **SF-36 GH** |  |  |  |  |  |
| *Intercept* | 5.591 | 5.200 | -4.601, 15.783 | 1.075 | .291 |
| *Baseline score* | .821 | .091 | .642, .999 | 9.023 | .00 |
| *Group: Group2* | 4.015 | 5.548 | -6.858, 14.888 | .724 | .474 |
| *Group: Group2 x Time: FollowUp* | -8.977 | 5.683 | -20.115, 2.162 | -1.580 | .125 |
| *Group: Group2 x Time: Week6* | -6.370 | 5.639 | -17.422, 4.681 | -1.130 | .267 |
| *Time: FollowUp* | .223 | 4.015 | -7.645, 8.092 | .056 | .956 |
| *Time: Week6* | 6.237 | 3.907 | -1.421, 13.896 | 1.596 | .120 |

*Notes.* Light grey shading indicates a medium effect size and dark grey shading indicates a large effect size*.* BPQ-SF=Body Perception Questionnaire–Short Form (ANS=Autonomic Nervous System, BA=Body Awareness); CGI-I=Clinical Global Impression-Improvement; FNSQ=Functional Neurological Symptom Questionnaire; GAD-7=Generalized Anxiety Disorder–7; MAIA-2=Multidimensional Assessment of Interoceptive Awareness (N=Noticing, ND=Not Distracting, NW=Not Worrying, AR=Attention Regulation, EA=Emotional Awareness, SR=Self Regulation, BL=Body Listening, T=Total); MDI=Multidimensional Dissociation Inventory (D=Detachment, DP=Depersonalisation, DR=Derealisation, MD=Memory Disturbance, EC=Emotional Constriction, ID=Identity Dissociation); PHQ-8=Patient Health Questionnaire–8; PHQ-15=Patient Health Questionnaire–15; SDQ-20=Somatoform Dissociation Questionnaire–20; SF-36=Short Form Health Survey–36 (PF=Physical Functioning, RLPH=Role Limitations Physical Health, RLEP=Role Limitations Emotional Problems, E/F=Energy/Fatigue, EW=Emotional Wellbeing, SF=Social Functioning, P=Pain, GH=General Health); TAS-20=Toronto Alexithymia Scale–20 (DDF=Difficulty Describing Feelings, DIF=Difficulty Identifying Feelings, EOT=Externally Oriented Thinking); TEC=Traumatic Experiences Checklist; WSAS=Work and Social Adjustment Scale.
